# Combining des-gamma-carboxyprothrombin and alpha-fetoprotein for hepatocellular carcinoma diagnosing: an update meta-analysis and validation study

**DOI:** 10.18632/oncotarget.20153

**Published:** 2017-08-07

**Authors:** Huaping Chen, Siyuan Chen, Shan Li, Zhijian Chen, Xuan Zhu, Meiyu Dai, Lingxi Kong, Xiaodan Lv, Zhili Huang, Xue Qin

**Affiliations:** ^1^ Department of Clinical Laboratory, First Affiliated Hospital of Guangxi Medical University, Nanning 530021, Guangxi, China

**Keywords:** des-gamma-carboxyprothrombin, AFP, hepatocellular cancer, diagnosis, meta-analysis

## Abstract

Controversies about the combination of des-gamma-carboxyprothrombin (DCP) and alpha-fetoprotein (AFP) for hepatocellular carcinoma diagnosing still exist. Hence, we performed this updated meta-analysis to estimate the diagnostic value of DCP , AFP and DCP + AFP in HCC. In addition, we conducted a validation study to analyze the performance of the candidate makers. After a systematic literature review, 27 studies from 20 articles were identified from four major databases. The pooled sensitivity and specificity were 69% and 89%, respectively, for DCP; for AFP, they were 65% and 88%, respectively; and they were 82% and 85%, respectively, for DCP + AFP. The values of the area under the curve (AUC) for DCP, AFP, DCP + AFP, respectively, were 0.88, 0.75, and 0.90. The validation study confirmed that the performance of DCP + AFP (sensitivity = 84%, specificity = 86%; AUC = 0.887) was higher than that of DCP (sensitivity = 76%, specificity = 92%; AUC = 0.843) or AFP (sensitivity = 73%, specificity = 92%; AUC = 0.837) alone.

## INTRODUCTION

Hepatocellular carcinoma (HCC) is one of the most (70–90%) frequent types of primary carcinoma of the liver and it is the third dominating cause of cancer-related mortality among men worldwide [1]. The incidence rate of HCC rises in accordance with increased rates of hepatitis C virus and hepatitis B virus infection [2]. Aflatoxin exposure, heavy alcohol drinking, nonalcoholic fatty liver disease, and smoking also contribute to the occurrence and progression of HCC [2].

Serum alpha-fetoprotein (AFP) is commonly used to diagnose HCC. However, owing to its low sensitivity and specificity, AFP testing alone is not recommended in diagnostic assessments of HCC [3–10]. Instead, it is used to combine with other serum or plasma tumor markers, which have been shown to have superior diagnostic abilities [3, 6, 11–15].

Des-gamma-carboxyprothrombin (DCP), an abnormal form of the prothrombin protein, is induced by a deficiency of vitamin K or antagonist-II [16]. The role of DCP as a biomarker of HCC was first reported in a study published 1984 [17], which found that DCP was present in 91% of HCC patients but not detectable in other benign liver diseases. Subsequently, numerous studies [6–8, 13, 18–20] demonstrated that a combined analysis of DCP and AFP led to better prediction of HCC although there are some controversies about the diagnostic accuracy of DCP + AFP.

Thus, we performed an update meta-analysis to contrast the diagnostic performance of DCP alone, AFP alone, and DCP + AFP in the detection of HCC. In addition, we performed a validation study of 45 HCC patients, 42 liver cirrhosis patients, 43 patients with hepatitis virus infections, and 44 normal controls to determine the diagnostic efficacy of these candidate markers.

## RESULTS

### Selection and characteristics of the included studies

**Figure 1:**
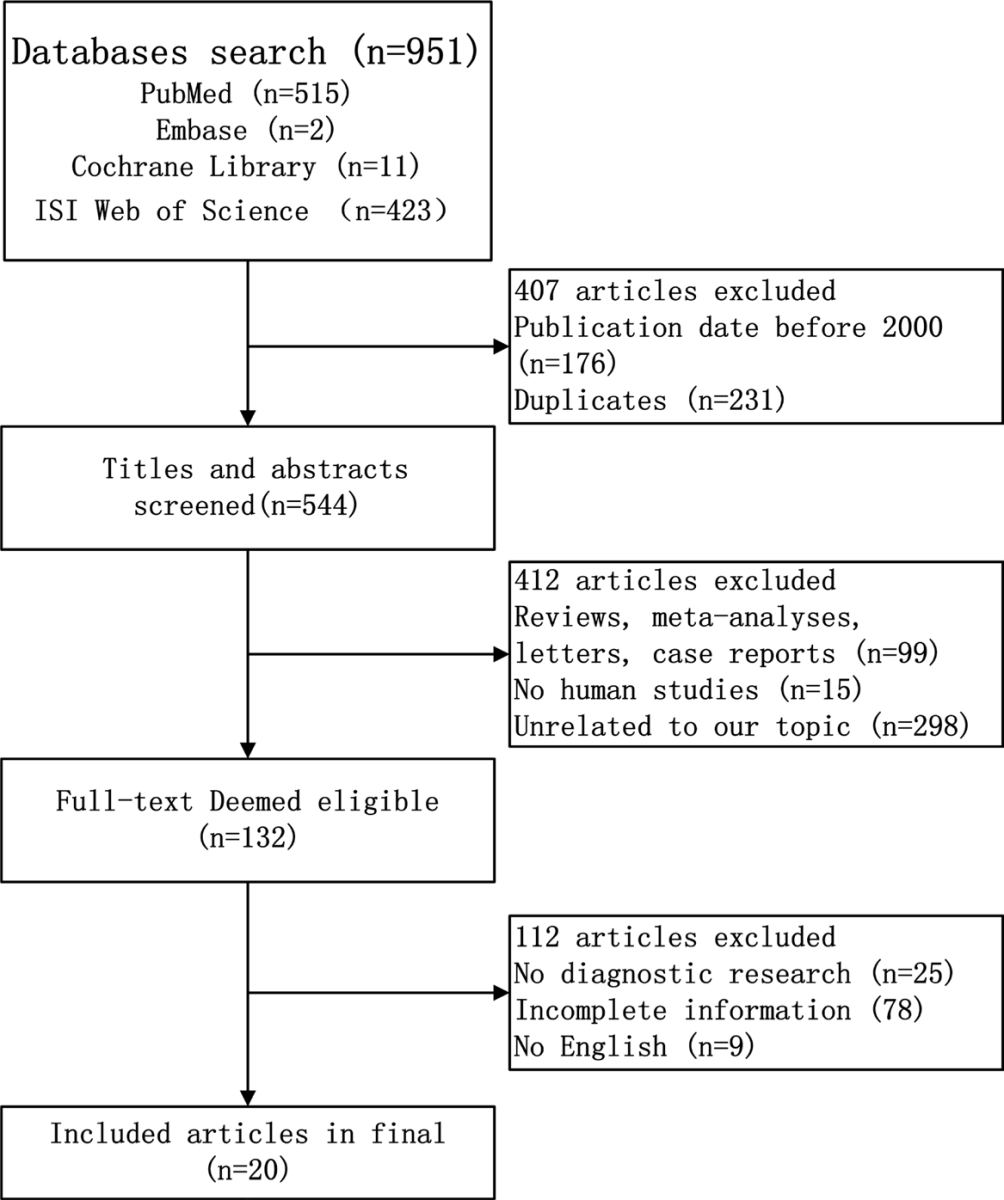
Flow diagram of study selection process for eligible studies

As shown in Figure 1, a search of four databases (PubMed, Embase, the Cochrane Library, and the ISI Web of Science) and a manual search identified 951 relevant articles. Of these, 176 were published before 2000, and 231 were duplicates. After sieving the titles and abstracts, 412 articles about reviews, case reports, letters, nonhuman studies, or unrelated to the topic were excluded. The rest of 132 studies were deemed to satisfactory, and they were read and evaluated carefully for a full-text review. Of these, 112 articles were eliminated for the following reasons: did not contain adequate data to construct a 2 *×* 2 table, did not contain sufficient information on the criteria used to diagnose HCC or analyzed only serum DCP and AFP alone, or they were published in non-English language periodicals. In total, 27 studies from 20 articles [11, 12, 14–16, 18–32] measured up for this meta-analysis and all of them were reported from 2000 to 2016.

This meta-analysis was included 7507 HCC patients and 5399 controls and the features of eligible studies were showed in Supplementary Table 1. All the included patients had undergone separate tests of serum and plasma levels of DCP and AFP as well as the test results of candidate markers (DCP, AFP, and DCP + AFP) had been analyzed by statistics.

The quality of the studies on the basis of the assessment scores of QUADAS [33–35] is summarized in Supplementary Table 2. Four articles met 10 of the 14 QUADAS norms, nine articles met 11 of the 14 QUADAS norms, six articles met 12 of the 14 QUADAS norms, and one article met 13 of the 14 QUADAS norms. According to the results of the QUADAS assessment, all the included studies were great in quality.

### Pooled diagnostic performance of DCP, AFP, DCP + AFP in HCC diagnosing

**Figure 2:**
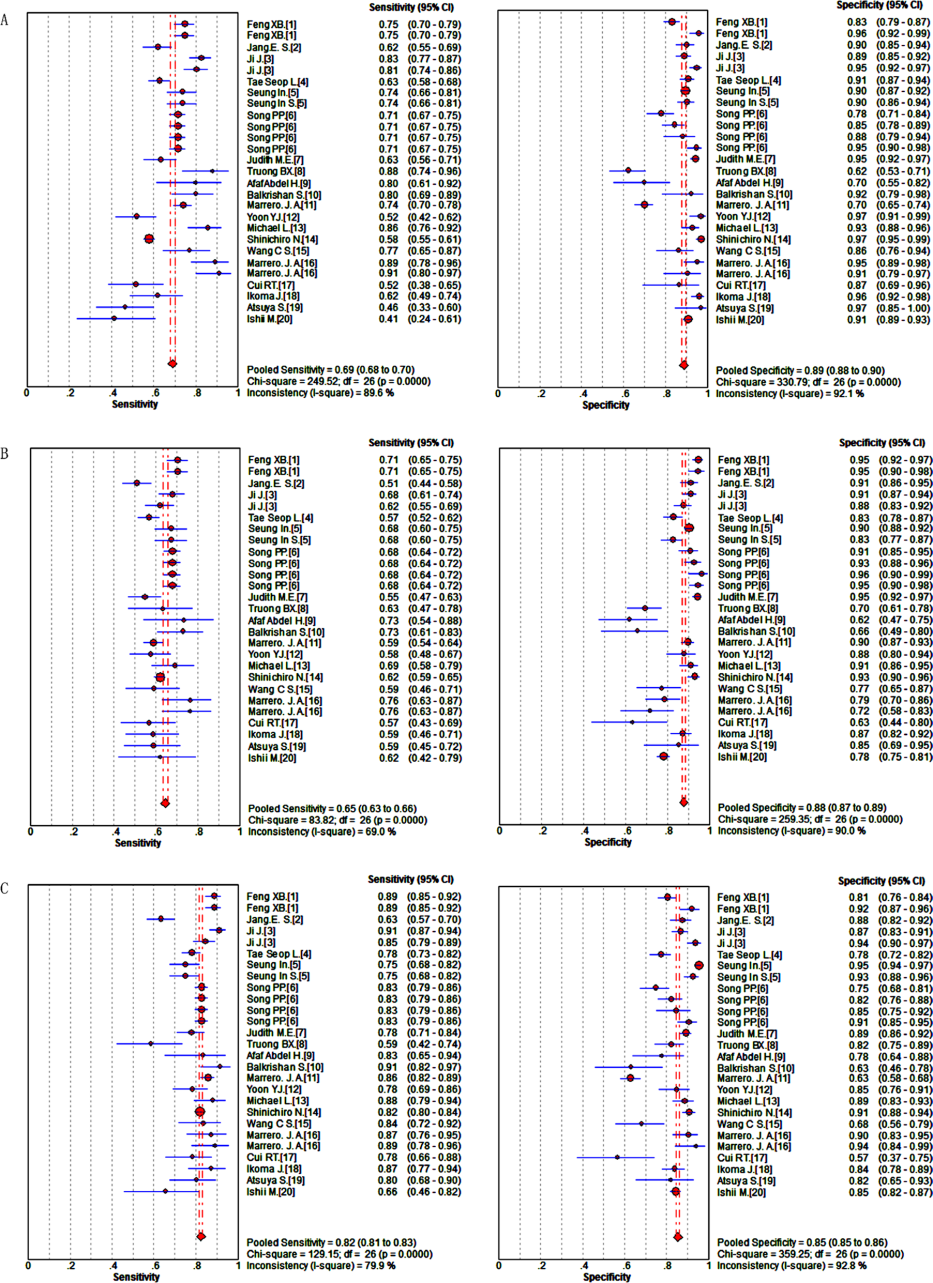
Forest plots of the sensitivity and specificity of candidate makers in the diagnosis of HCC (**A**) DCP; (**B**) AFP; (**C**) DCP + AFP.

Figure 2A–2C shows the sensitivity and specificity of the biomarkers: DCP: sensitivity (I^2^ = 89.6%, *P* = 0.0000) and specificity (I^2^ = 92.1%, *P* = 0.0000); AFP: sensitivity (I^2^ = 69.0%, *P* = 0.0000) and specificity (I^2^ = 90.0%, *P* = 0.0000); DCP + AFP: sensitivity (I^2^ = 79.9%, *P* = 0.0000) and specificity (I^2^ = 92.8%, *P* = 0.0000). As all the data pointed to significant heterogeneity in the meta-analysis, a random-effects model was chose. When the data were pooled, the sensitivity and specificity of DCP, AFP, and DCP + AFP were 0.69 (0.68–0.70) and 0.89 (0.88–0.90), 0.65 (0.63–0.66) and 0.88 (0.87–0.89), and 0.82 (0.81–0.83) and 0.85 (0.85–0.86), respectively.

**Figure 3:**
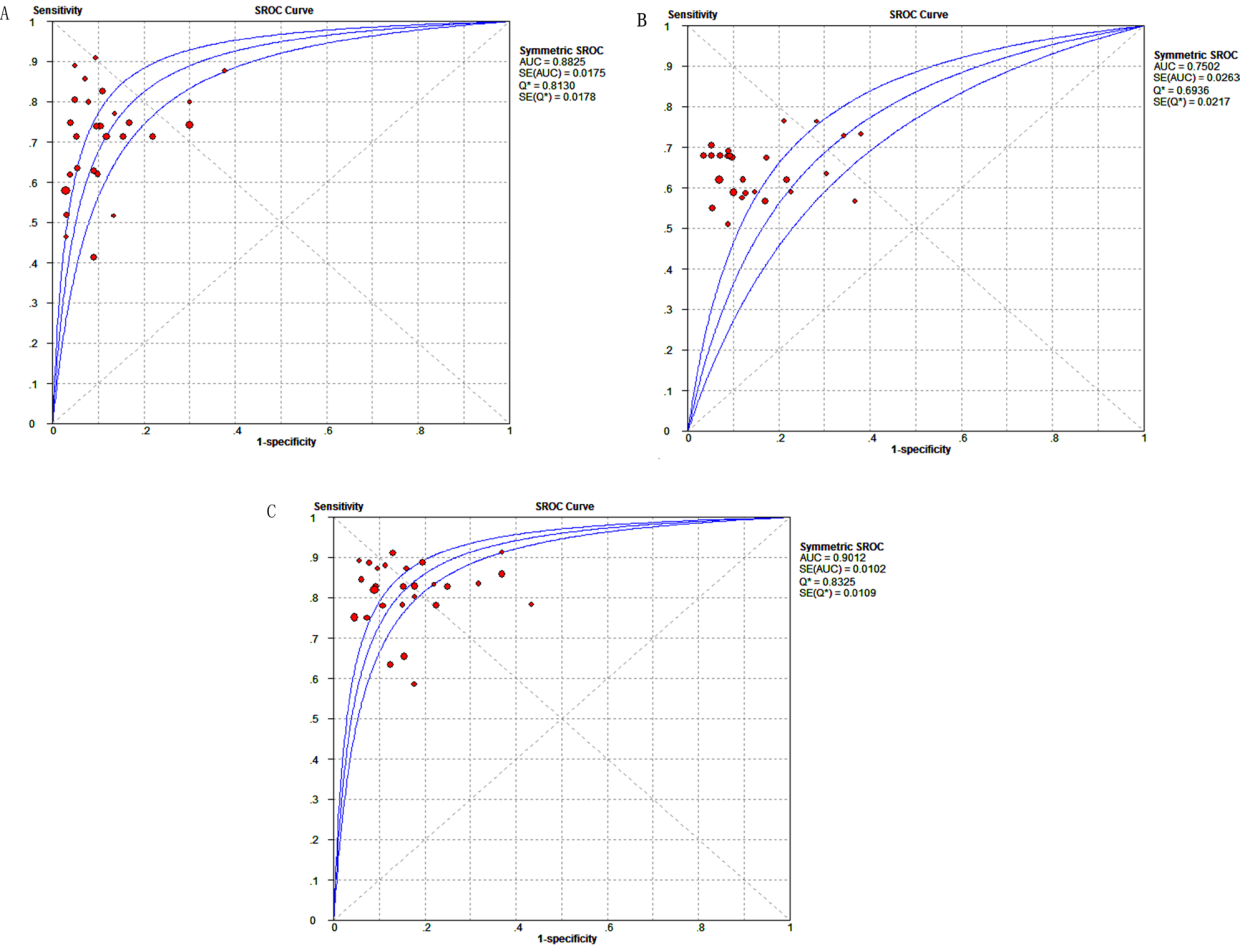
SROC curve of candidate makers for diagnosing HCC (**A**) DCP; (**B**) AFP; (**C**) DCP + AFP.

The summary receiver operator characteristic (SROC) curves of DCP, AFP, DCP + AFP are shown in Figures 3A–3C. The results demonstrated that the area under the curve (AUC) values for DCP, AFP, DCP + AFP were, respectively, 0.88, 0.75, and 0.90. The pooled positive likelihood ratios (PLRs), negative likelihood ratios (NLRs), and diagnostic odds ratios (DORs) were 7.28 (5.52–9.61), 0.33 (0.29–0.37), 24.59 (17.98–33.62), respectively, for DCP; 5.07 (3.98–6.45), 0.41 (0.38–0.44), and 12.96 (9.90–16.98), respectively, for AFP; and 5.48 (4.31–6.96), 0.22 (0.19–0.25), and 26.45 (19.61–35.68), respectively, for DCP + AFP. The diagnostic performance of the candidate biomarkers are presented in Table 1. The results suggested that the diagnostic value of DCP + AFP in the detection of HCC was better than that of either DCP or AFP alone.

**Table 1:** Meta-analysis results of DCP, AFP, DCP+AFP

Maker	Se(95%CI)I^2^	Sp(95%CI)I^2^	PLR(95%CI)I^2^	NLR(95%CI)I^2^	DOR(95%CI)I^2^	AUC
DCP	0.690.68-0.70)89.6%	0.89(0.88-0.90)92.1%	7.28(5.52-9.61)91.6%	0.33(0.29-0.37)87.4%	24.59(17.98-33.62)84.2%	0.88
AFP	0.65(0.63-0.66)69.0%	0.88(0.87-0.90)90.0%	5.07(3.98-6.45)89.0%	0.41(0.38-0.44)75.2%	12.96(9.90-16.98)81.2%	0.75
DCP+AFP	0.82(0.81-0.83)79.9%	0.85(0.85-0.86)92.8%	5.48(4.31-6.96)92.3%	0.22(0.19-0.25)81.6%	26.45(19.61-35.68)84.9%	0.90

Se: sensitivity, Sp: specificity, PLR: positive likelihood ratio, NLR: negative likelihood ratio, DOR: diagnostic odds ratio, CI: confidence interval, AUC: area under ROC curve.

### Threshold effects and meta-regression analysis of heterogeneity

To identify potential sources of heterogeneity, tests for threshold effects were conducted by Meta-Disc software. Spearman's correlation coefficient value was 0.295 (*P* = 0.135) for DCP. It was −0.007 (*P* = 0.971) and 0.060 (*P* = 0.765) for AFP and DCP + AFP, respectively, which suggested there were no threshold effects in this meta-analysis.

Other than threshold effects, the diversity of study populations in different countries, methodology used, and size of the study population can be potential sources of heterogeneity. To analyze these factors, a meta-regression was performed (Table 2). With regard to DCP, the results revealed no significant heterogeneity in respect of country (coefficient = −0.132, *P* = 0.1736), methodology (coefficient = 0.044, *P* = 0.7654), or size of the study population (coefficient = 0.000, *P* = 0.2863). It proved other factors might lead to the high heterogeneity of DCP. For AFP, they showed no significant heterogeneity in respect of the size of the study population (coefficient = −0.001, *P* = 0.0525). Interestingly, the results of the meta-regression indicated that differences in countries (coefficient = −0.088, *P* = 0.0356) and methodology (coefficient = −0.106, *P* = 0.0454) might be the source of heterogeneity in AFP studies.

**Table 2:** Meta-regression analyses of the heterogeneity in DCP and AFP

Variable	DCP					AFP				
	Coeff.	Sth.Err.	P-value	RDOR	(95%)CI	Coeff.	Sth.Err.	P-value	RDOR	(95%)CI
Country	−0.132	0.0940	0.1736	0.88	(0.72; 1.06)	−0.088	0.0394	0.0356	0.92	(0.84; 0.99)
Method	0.044	0.1454	0.7654	1.04	(0.77; 1.41)	−0.106	0.0454	0.0291	0.90	(0.82; 0.99)
Population	0.000	0.0004	0.2863	1.00	(1.00; 1.00)	0.001	0.0003	0.0525	1.00	(1.00; 1.00)

Std.Err: standard error, RDOR: ratio of diagnostic odds ratio, CI: confidence interval.

### Sensitivity analysis and publication bias

Removal of individual studies included in this meta-analysis was evaluated each time to determine the impact of the remaining data set on the sensitivity and specificity. The merged results were stable, and they were not altered substantially by individual studies.

**Figure 4:**
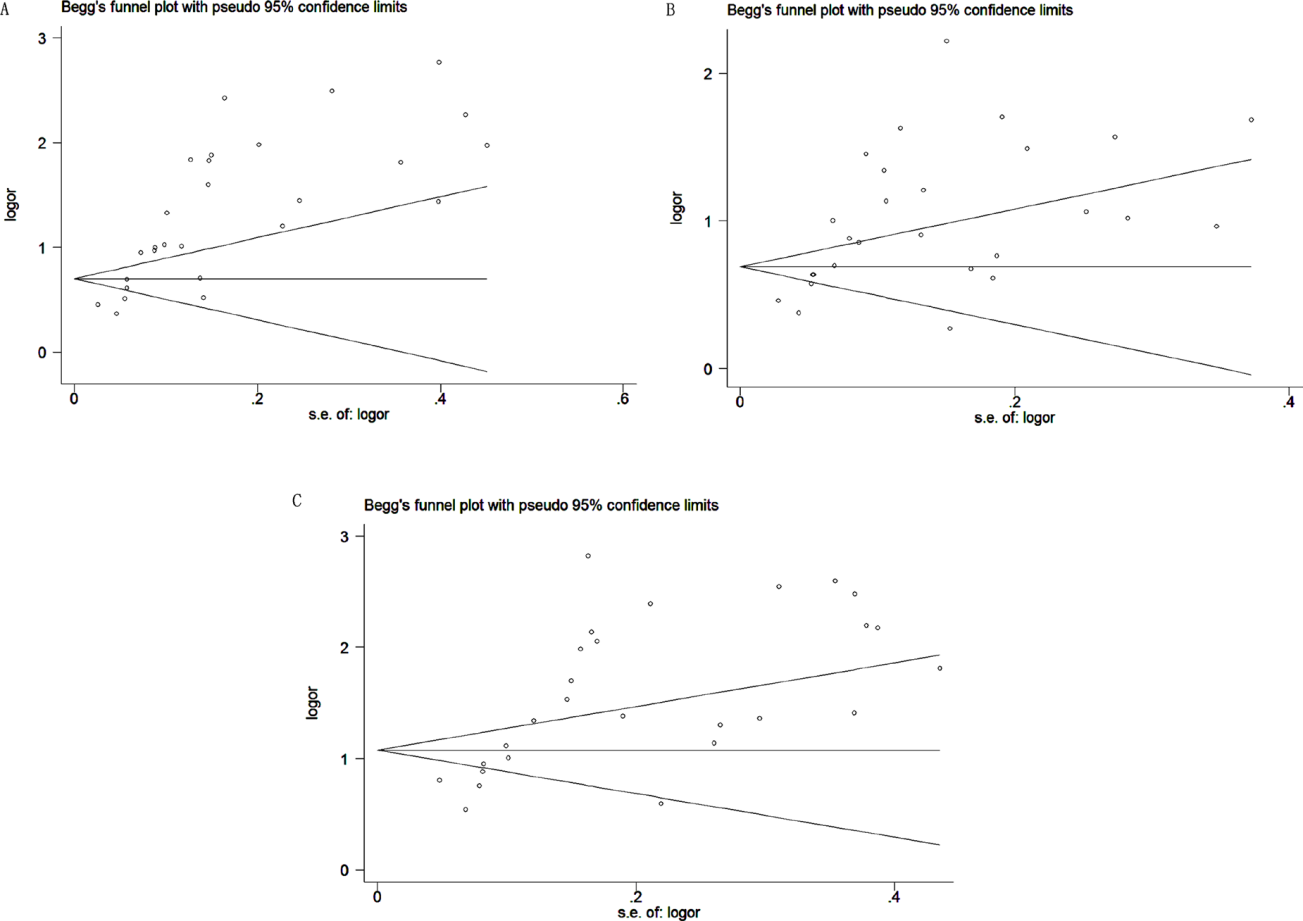
Begg's funnel plot of candidate makers for the studies included in the meta-analysis (**A**) DCP; (**B**) AFP; (**C**) DCP+AFP.

As shown in Figure 4A–4C, Begg's funnel plot asymmetry pointed to potential publication bias in the included studies. Thus, a quantitative Egger's linear regression test was conducted to provide statistic evidence of funnel plot symmetry or asymmetry. The results pointed to potential publication biases were detected in DCP (*P* = 0.000), AFP (*P* = 0.000) and DCP + AFP (*P* = 0.000) studies.

### Validation study of the diagnostic performance of DCP + AFP in HCC

**Figure 5:**
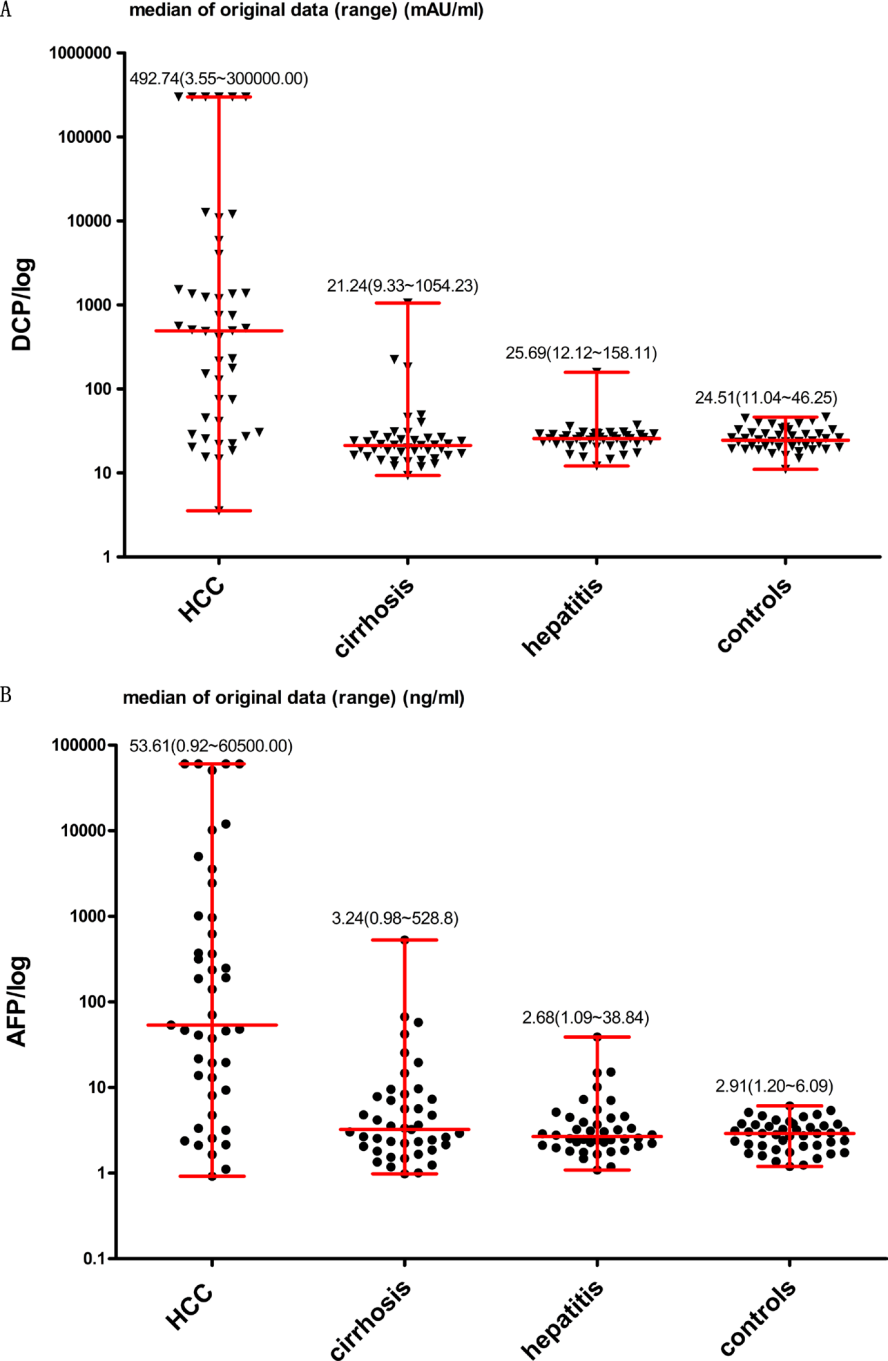
Levels of DCP and AFP in four different groups DCP was measured in mAU/ml and AFP in ng/ml. Median of original data (minimum to maximum) was shown respectively in the figures.

In the present study, there were 45 HCC patients (G 1), 43 liver cirrhosis patients (G 2), 42 patients with hepatitis virus infections (G 3), and 44 healthy individuals (G 4) (Supplementary Table 3). There was no statistically significance difference in the sex of the individuals in the four groups (*P* = 0.311). There was no statistic difference in the age of the patients in the different groups: G 1 and G 2 (*P* = 0.386), G 1 and G 3 (*P* = 0.410), G 1 and G 4 (*P* = 0.709), G 2 and G 3 (*P* = 1.000), G 2 and G 4 (*P* = 0.328), G 3 and G 4 (*P* = 0.289). As shown in Figure 5 (A, B), the serum levels of both DCP and AFP were observably higher in G 1 than in G 2 (*P* = 0.000 for both). They were also observably higher in G 1 compared to that in G 3 (*P* = 0.000 for both) and meanwhile in G 1 than G 4 (*P* = 0.000 for both). As shown in Supplementary Figure 1, the AUC values of DCP, AFP, and DCP + AFP in distinguishing between HCC (G 1) and liver cirrhosis (G 2) were 0.860 (95% CI: 0.770–0.925, sensitivity [Se]: 0.76, specificity [Sp]: 0.86), 0.805 (95% CI: 0.707–0.882, Se: 0.73, Sp: 0.84), and 0.859 (95% CI: 0.768–0.924, Se: 0.84, Sp: 0.77), respectively. In distinguishing HCC (G 1) from non-HCC (G 2, 3, and 4), the AUC values of DCP, AFP, and DCP + AFP were 0.843 (95% CI: 0.781–0.894, Se: 0.76, Sp: 0.92), 0.837 (95% CI: 0.774–0.889, Se: 0.73, Sp: 0.92), and 0.887 (95% CI: 0.830–0.930, Se: 0.84, Sp: 0.86), respectively (Table 3).

**Table 3:** Diagnostic values of candidate makers for hepatocellular carcinoma

Makers	AUC	(95% CI)	Cut-off value	Se	Sp	Youden index
DCP						
HCC vs. cirrhosis	0.860	0.770–0.925	40 mAU/ml	0.76	0.86	0.62
HCC vs. no-HCC	0.843	0.781–0.894		0.76	0.92	0.67
AFP						
HCC vs. cirrhosis	0.805	0.707–0.882	11 ng/ml	0.73	0.84	0.57
HCC vs. no-HCC	0.837	0.774–0.889		0.73	0.92	0.66
DCP+AFP						
HCC vs. cirrhosis	0.859	0.768–0.924	-	0.84	0.77	0.61
HCC vs. no-HCC	0.887	0.830–0.930	-	0.84	0.86	0.70

AUC: area under the curve; CI: confidence interval; Se: sensitivity; Sp: specificity.

## DISCUSSION

The results of this meta-analysis including 27 studies from 20 articles demonstrated that the overall sensitivity and specificity of DCP was, respectively, 69% and 89%. For AFP, the overall sensitivity and specificity was 65% and 88%. For DCP + AFP, these values were 82% and 85%, respectively. Although the specificity of the biomarkers did not differ greatly, the sensitivity of DCP + AFP was markedly higher, signifying the superior diagnostic value of DCP + AFP in HCC. DOR is a specific value of PLR and NLR. It is a single indicator of the performance of a trial but independent of prevalence. DOR ranges from zero to infinity, and higher values suggest preferable diagnostic performance. In the present study, the diagnostic odds ratio values of DCP, AFP, and DCP + AFP (24.59 vs. 12.96 vs. 26.45) showed that DCP + AFP was better as the indicator of HCC than either DCP or AFP alone. Furthermore, the AUC values of DCP, AFP, and DCP + AFP (0.88 vs. 0.75 vs. 0.90) indicated that the accuracy of DCP + AFP in HCC diagnosing was better than that of AFP alone. However, in the findings of the meta-analysis, DCP + AFP were not superior fully to DCP, as shown by the AUC values (0.90 vs. 0.88).

Heterogeneity refers to variation in or between studies in systematic reviews. In the present study, the I^2^ test revealed considerable heterogeneity in DCP, AFP, and DCP + AFP. However, Spearman's correlation coefficient values of DCP, AFP, and DCP + AFP indicated that heterogeneity was caused by no-threshold effects. Thus, we performed a meta-regression, a quantitative method to inspect the impact of variables, such as the size of the study population, to acquire more information about the source of the heterogeneity. The results of the meta-regression showed that differences in test methods and sample size for AFP may contribute to the observed heterogeneity in the application of AFP in HCC detection but not in the application of DCP. The another factor (study population from different countries) analyzed in the meta-regression also could not explain the heterogeneity in the performance of DCP. Therefore, the results of the meta-regression were unable to convincingly explain the heterogeneity in this meta-analysis. In addition, exploration of the potential role of different types of specimens (serum or plasma) or diverse issues of publications of DCP and AFP studies in the meta-regression showed that these sources did not contribute to the observed heterogeneity (Supplementary Table 4).

In addition to heterogeneity, publication bias was another limitation in the present study. We executed a sensitivity analysis to determine the stability and reliability of the overall data. Removing each of the 27 studies at a time, the pooled sensitivity varied from 0.68 (0.67 to 0.70) to 0.72 (0.70 to 0.73) for DCP, 0.64 (0.63 to 0.65) to 0.65 (0.63 to 0.66) for AFP, and 0.82 (0.81 to 0.83) to 0.83 (0.82 to 0.84) for DCP + AFP. The pooled specificity ranged from 0.88 (0.87 to 0.89) to 0.90 (0.89 to 0.91) for DCP, 0.87 (0.86 to 0.88) to 0.89 (0.88 to 0.90) for AFP, and 0.84 (0.83 to 0.85) to 0.87 (0.86 to 0.88) for DCP + AFP. Thus, sensitivity analyses in this meta-analysis illuminated that the pooled result and conclusions did not fluctuate by the various decisions that could be made during articles reviewing. It signifies the results of our meta-analysis can be considered with a higher credibility. The results of Egger's linear regression test of DCP, AFP and DCP + AFP in HCC diagnosing were consistent with Begg's funnel plots, which demonstrated that publication bias may be present in this meta-analysis. In the present study, we used an optimal search strategy, which included a comprehensive search of four English databases and a manual search to ensure that no relevant articles were overlooked. However, studies with negative results may remain unpublished, with both authors and editors tending to publish positive results of which these two inestimable reasons may lead to publication bias in this article. Therefore, we further performed the tirm and fill method to adjust for publication bias in the present meta-analysis [36]. For DCP in HCC diagnosing studies, the overall analysis merging the theoretical 33 (27 + 6) studies had little variance with the unredressed 27 studies (variance = 0.217, *P* = 0.000 vs. variance = 0.230, *P* = 0.000) and that was similar to DCP + AFP studies (variance = 0.297, *P* = 0.000 vs. variance = 0.341, *P* = 0.000); for AFP researches, none hypothetical study was added and no data were changed between adjusted and unadjusted ones (variance = 0.135, *P* = 0.000 for both) (Supplementary Figure 2). All of these results implied that the absent articles with negative results did not influence significantly the overall accuracy in our meta-analysis.

In our literature search, there are three similar meta-analyses that also assessed DCP, AFP, and DCP + AFP for HCC diagnosing [3–5]. Compared with the previous studies [3–5], the advantages of the present meta-analysis are as follows: First, this meta-analysis evaluated the diagnostic performance of DCP + AFP, whereas the previous meta-analyses focused on multiple serum biomarkers for HCC. The present meta-analysis is less ambiguous than previous ones. Second, this meta-analysis included more studies on DCP and AFP and larger samples sizes than previous studies. Third, all the included HCC cases were diagnosed according to gold standard methods to further enhance the validity of the findings. Fourth, the data on DCP and AFP were derived from identical groups to strengthen the comparability of the diagnostic accuracy of the candidate markers (DCP, AFP, and DCP + AFP). Fifth, we included a number of factors (study populations from different countries, test methods, and sample size) not included in previous meta-analyses to explore the source of heterogeneity. Besides, the major orientation in our meta-analysis were similar with a systematic review [37] published in 2014 but different in topics. The present meta-analysis was aimed at the diagnostic performance of DCP + AFP when another aimed to compare the deference between DCP and AFP as biomarkers in diagnosing HCC. In that review [37], the overall evaluations were: sensitivity = 0.63, specificity = 0.91, AUC = 0.83 for DCP, sensitivity = 0.59, specificity = 0.86, AUC = 0.77 for AFP, sensitivity = 0.81, specificity = 0.83, AUC = 0.88 for DCP + AFP. All of the summary results in our meta-analysis were agreed with that in that review. Comparing the systematic review [37], the merits of our meta-analysis were listed below: First, the strict inclusive and exclusive criteria were constituted in this meta-analysis, an English-language restriction and included HCC patients must be diagnosed by histology, for instance. Second, high quality was performed in each of included articles according to evaluated tool but the precious review was not. Finally, we conducted the analyses of threshold effects and meta- regression to explore the heterogeneity.

In our validation study, we evaluated the serum levels of the candidate markers in four different groups (HCC patients, *n* = 45; liver cirrhosis patients, *n* = 43; patients with the infections of hepatitis virus, *n* = 42; and healthy controls, *n* = 44). The results of the validation study demonstrated that “DCP + AFP” was a superior marker to DCP or AFP, which was in accordance with the results of our meta-analysis. The AUC value of DCP + AFP was no significantly higher than that in DCP or AFP (0.860 vs. 0.805 vs. 0.859) for distinguishing HCC from liver cirrhosis. As the analysis focus on individuals with no-HCC, the ROC curve and AUC value showed better diagnostic efficiency of DCP + AFP as compared to that of DCP (0.887 vs. 0.843) or AFP (0.887 vs. 0.837) in distinguishing HCC from non-HCC. The conclusions of our validation study are also in accordance with those of the majority of studies [11–14, 38–40]. However, the smaller sample number is a limitation of our validation study.

Based on the results of our meta-analysis and validation study, the diagnostic performance of a combination of DCP + AFP is prominent to that of DCP or AFP alone in the detection of HCC. Further research with optimized designs, greater numbers of studies, and larger sample sizes are required to shed light on remaining inconsistencies in variant results of combining des-gamma-carboxyprothrombin and alpha-fetoprotein for hepatocellular carcinoma diagnosing.

## MATERIALS AND METHODS

### Search strategy

A systematic literature search was conducted for relevant articles published in PubMed, Excerpta Medica Database (Embase), the Cochrane Library, and ISI Web of Science before 27 December 2016. For AFP, the search terms were AFP and α-fetoprotein. For DCP, the search terms were acarboxyprothrombin, PIVKA II, DCP, decarboxyprothrombin, non-carboxylated factor II, protein induced by vitamin K absence, and antagonists. For HCC, the search terms were liver neoplasms, hepatic neoplasms, hepatocellular cancer, liver cell carcinomas, HCC, small hepatocellular carcinoma, and SHCC. Moreover, the references lists of review articles were searched manually to ascertain more relevant studies.

### Eligibility criteria and study selection

To be involved in the meta-analysis, all the articles must fulfill the criteria as follows: (i) contain both individual and combined sensitivity and specificity data on serum or plasma DCP and AFP assays, (ii) consist of patients with a pathologically proven diagnosis of HCC. The exclusion criteria were as follows: (i) reviews, meta-analyses, case reports, or letters; (ii) nonhuman studies; (iii) unrelated to the topic; (iv) incomplete information; and (v) non-English. When study populations overlapped, only the largest sized study or most comprehensive study was incorporated in the concluding analysis.

### Data extraction and quality assessment

The following information was collected from each paper: the name of author, year of publication, country of research, quantity of cases with HCC, quantity of contrasts which may included healthy individuals, patients with hepatitis, cirrhosis or other nonmalignant hepatopathy and non-HCC cancers, assay methods, cut-off points, and the sensitivity and specificity of DCP and AFP alone and their combination (DCP + AFP). The QUADAS (Quality Assessment of Diagnostic Accuracy Studies) tool [33–35] was applied to evaluate the quality of the contained studies. Using the 14-item tool, the evaluators rated each item as “Yes”, “No,” or “Unclear.” The reviewers assigned a mark of “1” for “Yes” and “0” for “No” or “Unclear” to each of the 14 items. A high-quality article was deemed one that attained a final Q score of 10 or more.

### Validation study of the diagnostic value of DCP, AFP, and DCP + AFP in diagnosing HCC

The study consisted of HCC patients (G 1, *n* = 45), liver cirrhosis patients (G 2, *n* = 43), patients with hepatitis virus infections (G 3, *n* = 42), and healthy controls (G 4, *n* = 44) matched in sex and age from the First Affiliated Hospital of Guangxi Medical University. HCC was diagnosed based on two or more typical imaging modalities or histological examinations. Cirrhosis patients were excluded if they showed evidence of progression to HCC. Patients with hepatitis virus infections were determined by laboratory diagnoses. Healthy subjects who tested negative results of hepatitis B virus surface antigen and with the normal levels of both of aspartate transaminase and alanine transaminase, were included. Individuals with obstructive jaundice and those taking warfarin, as well as samples with hemolysis and lipemia, were also excluded.

Serum levels of DCP were determined using a chemiluminescent immunoassay assay, and AFP concentrations were resolved by electrochemiluminescence immunoassay, according to the manufacturer's instructions.

### Statistical Analysis

### Meta-analysis

Heterogeneity was evaluated by the I^2^ test. A value of *P* < 0.1 and I^2^ > 50% were considered indicative of significant heterogeneity. A random effects model was selected in cases of obvious heterogeneity (*P* < 0.1, I^2^ > 50%). Otherwise (i.e., in cases of *P* ≥ 0.1, I^2^ ≤ 50%), a fixed effects model was applied. To evaluate the performance of the diagnostic studies, Spearman's correlation coefficient was applied to test for threshold effects and the overall sensitivity, specificity, likelihood ratios, and diagnostic odds ratios (DORs), together with their 95% confidence intervals (CIs), in addition to their summary receiver operating characteristic (SROCs) curves and area under the curve (AUCs) were calculated by the quantities of true positive, false positive, false negative, and true negative. Furthermore, a meta-regression analysis, sensitivity analysis, and analyses for publication bias were executed simultaneously. All the statistic analyses were conducted utilizing Meta-Disc software (version 1.4) and Stata (version 12.0).

### Validation study

In the validation study, the statistical data analyses were performed with IBM SPSS (version 20), and sectional graphics were executed using GraphPad Prism (version 5.01). A chi-square test was applied to compare classified variables. The Mann–Whitney test was performed to compare differences between two groups. A two-tailed test of *P*-value < 0.05 was considered as statistical differences. ROC curve and binary logistic regression analyses were used to calculate the areas under the ROC (AUROC) of DCP + AFP. The AUC was served for comparing the performance of DCP, AFP, and DCP + AFP. The cutoff values for DCP and AFP were 40mAU/ml and 11ng/ml, in accordance with the manufacturer's instructions. As hepatic cirrhosis is known to be a pathogenic factor that is closely related to HCC, in this study, we analyzed the AUC, sensitivity, specificity, and Youden index in patients with and without HCC (G 1 vs. G 2, 3, and 4) and in HCC patients and liver cirrhosis patients (G 1 vs. G 2).

## SUPPLEMENTARY MATERIALS FIGURES AND TABLES







## Abbreviations

DCP: des-gamma-carboxyprothrombin
AFP: alpha-fetoprotein
HCC: Hepatocellular carcinoma
AUC: area under the curve
QUADAS: Quality Assessment of Diagnostic Accuracy Studies
Se: sensitivity
Sp: specificity
PLRs: positive likelihood ratios
NLRs: negative likelihood ratios
DORs: diagnostic odds ratios
CIs: confidence intervals
