# Supplementary material for: Combining des-gamma-carboxyprothrombin and alpha-fetoprotein for hepatocellular carcinoma diagnosing: an update meta-analysis and validation study
Source: Oncotarget. 2017 Aug 7;8(52):90390–401. doi: 10.18632/oncotarget.20153 (PMC5685759; doi:10.18632/oncotarget.20153)
Supplement: Supplementary file 1 [file oncotarget-08-90390-s001.pdf]

Combining des-gamma-carboxyprothrombin and alpha-fetoprotein for hepatocellular carcinoma diagnosing: an update meta-analysis and validation study

SUPPLEMENTARY MATERIALS

Supplementary Table 1: Essential characteristics of 27 included studies. See Supplementary_Table_1

Supplementary Table 2: QUADAS assessment of included articles. See Supplementary_Table_2

Supplementary Table 3: Simple statistics based on original data

Group	Case number	DCP		AFP		DCP+AFP	
		Positive	Negative	Positive	Negative	Positive	Negative
Controls	44	4	40	0	44	4	40
Hepatitis	42	1	41	3	39	4	38
Cirrhosis	43	5	38	7	36	10	33
HCC	45	34	11	33	12	41	4

Supplementary Table 4: Supplementary meta-regression analyses of the heterogeneity in DCP and AFP

Variable	DCP					AFP				
	Coeff.	Std.Err.	*P*-value	RDOR	(95%)CI	Coeff.	Std.Err.	*P*-value	RDOR	(95%)CI
Sample	0.336	0.4137	0.4247	1.40	(0.59; 3.29)	1.006	0.5031	0.0575	2.73	(0.97; 7.74)
Year	0.008	0.0317	0.7961	1.01	(0.94; 1.08)	0.0030	0.0204	0.1501	1.03	(0.99; 1.08)

Std.Err: standard error, RDOR: ratio of diagnostic odds ratio, CI: confidence interval.

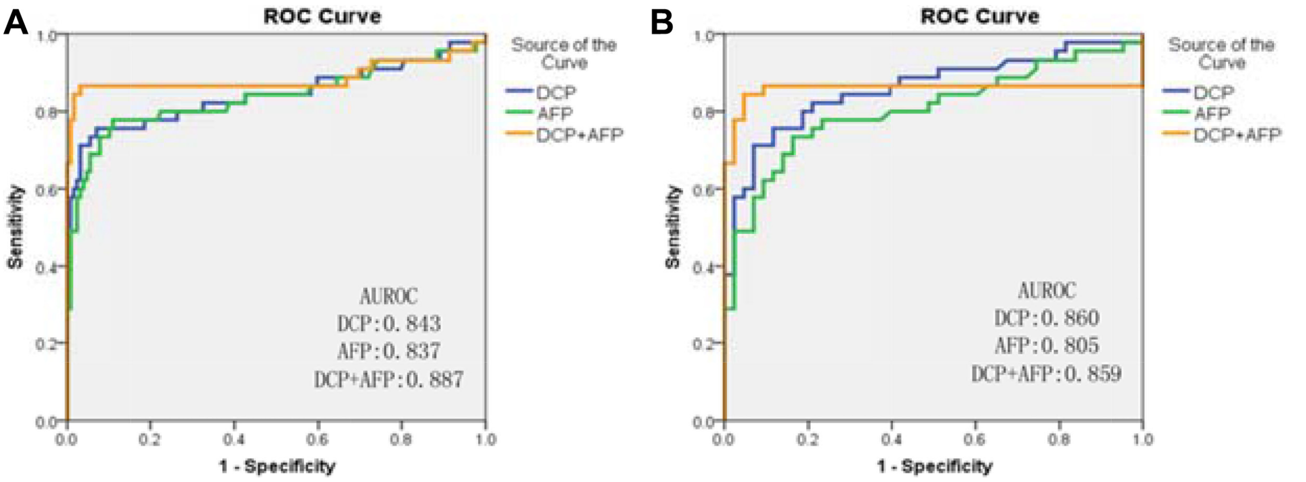

Supplementary Figure 1: ROC curves for DCP, AFP and DCP + AFP in diagnosing HCC. (**A**) HCC vs. no-HCC; (**B**) HCC vs. Cirrhosis.

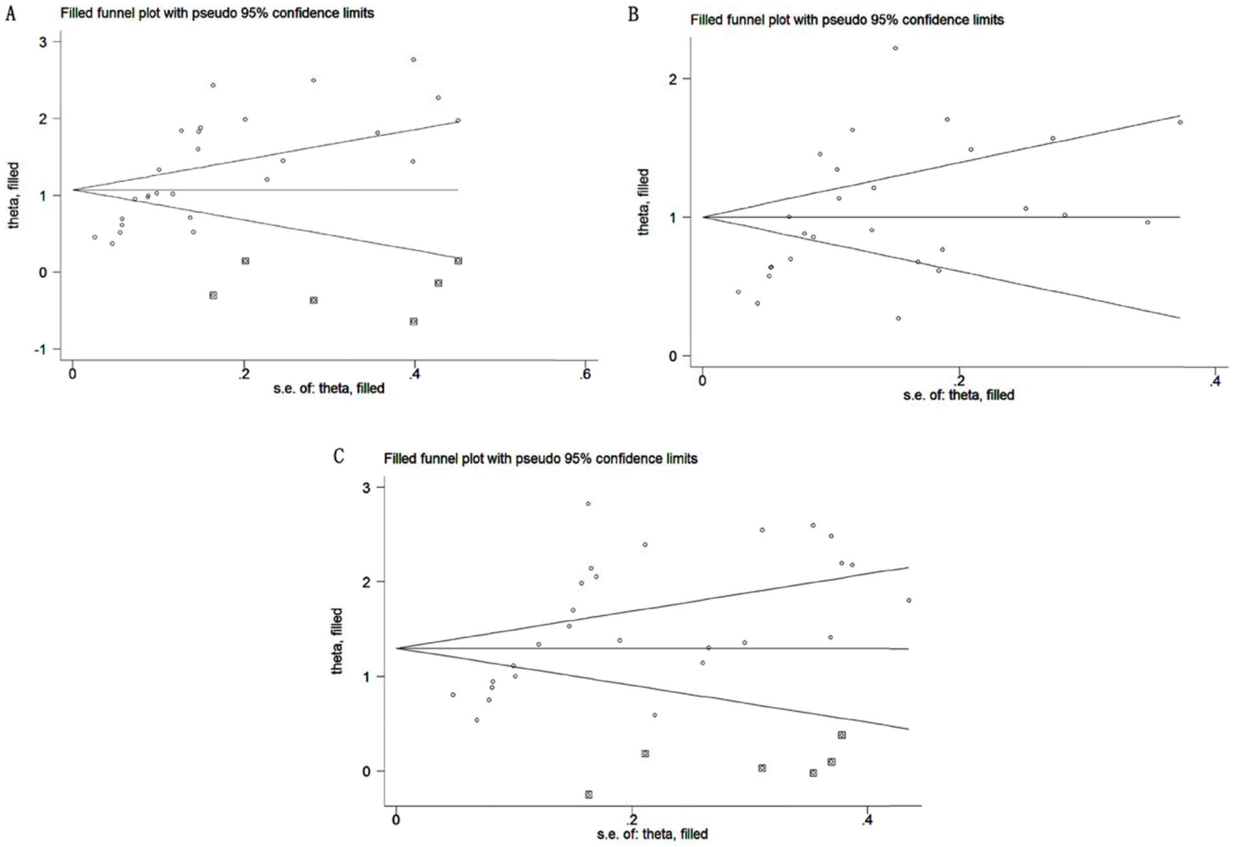

Supplementary Figure 2: Funnel plot for estimated missing studies after adjustment for publication bias utilizing trim and fill method. Hollow circle in box shows the estimated missing studies. (**A**) DCP; (**B**) AFP; and (**C**) DCP + AFP.
