# Supplementary material for: Combining des-gamma-carboxyprothrombin and alpha-fetoprotein for hepatocellular carcinoma diagnosing: an update meta-analysis and validation study
Source: Oncotarget. 2017 Aug 7;8(52):90390–401. doi: 10.18632/oncotarget.20153 (PMC5685759; doi:10.18632/oncotarget.20153)
Supplement: Supplementary file 3 [file oncotarget-08-90390-s003.docx]

| **Author** | **Item 1** | **Item 2** | **Item 3** | **Item 4** | **Item 5** | **Item 6** | **Item 7** | **Item 8** | **Item 9** | **Item 10** | **Item 11** | **Item 12** | **Item 13** | **Item 14** | **Q** |
| --- | --- | --- | --- | --- | --- | --- | --- | --- | --- | --- | --- | --- | --- | --- | --- |
| Feng XB.[[1](#_ENREF_1)] | Y | Y | Y | U | Y | Y | U | Y | Y | N | Y | Y | Y | U | 10 |
| Jang.E. S.[[2](#_ENREF_2)] | Y | Y | Y | U | Y | Y | Y | Y | Y | N | Y | Y | U | Y | 11 |
| Ji J.[[3](#_ENREF_3)] | Y | Y | Y | U | Y | Y | N | Y | Y | N | Y | Y | Y | Y | 11 |
| Tae Seop L.[[4](#_ENREF_4)] | Y | Y | Y | U | Y | Y | Y | Y | Y | N | Y | Y | U | Y | 11 |
| Seung In.[[5](#_ENREF_5)] | Y | Y | Y | Y | Y | Y | Y | Y | Y | N | Y | Y | Y | U | 12 |
| Song PP.[[6](#_ENREF_6)] | Y | Y | Y | U | U | U | Y | Y | Y | N | Y | Y | Y | Y | 10 |
| Judith M.E.[[7](#_ENREF_7)] | Y | N | Y | U | Y | Y | Y | Y | Y | N | Y | Y | Y | Y | 11 |
| Truong BX.[[8](#_ENREF_8)] | Y | Y | Y | U | Y | Y | Y | Y | Y | N | Y | Y | Y | Y | 12 |
| Afaf Abdel H.[[9](#_ENREF_9)] | Y | Y | Y | U | Y | Y | Y | Y | Y | N | Y | Y | Y | U | 11 |
| Balkrishan S.[[10](#_ENREF_10)] | Y | Y | Y | U | Y | Y | Y | Y | Y | N | Y | Y | Y | Y | 12 |
| Marrero. J. A.[[11](#_ENREF_11)] | Y | Y | Y | Y | Y | Y | U | Y | Y | Y | Y | Y | Y | Y | 13 |
| Yoon YJ.[[12](#_ENREF_12)] | Y | U | Y | Y | Y | Y | N | Y | Y | N | N | Y | Y | Y | 10 |
| Michael L.[[13](#_ENREF_13)] | Y | U | Y | Y | Y | Y | U | Y | Y | N | Y | Y | Y | Y | 11 |
| Shinichiro N.[[14](#_ENREF_14)] | Y | Y | Y | Y | Y | Y | N | Y | Y | N | Y | Y | Y | Y | 12 |
| Wang C S.[[15](#_ENREF_15)] | Y | Y | Y | U | Y | Y | Y | Y | Y | N | Y | Y | Y | Y | 12 |
| Marrero. J. A.[[16](#_ENREF_16)] | Y | Y | Y | U | Y | Y | Y | Y | Y | N | Y | Y | Y | U | 11 |
| Cui RT.[[17](#_ENREF_17)] | Y | Y | Y | Y | Y | Y | Y | Y | Y | N | Y | Y | N | U | 11 |
| Ikoma J.[[18](#_ENREF_18)] | Y | U | Y | Y | U | Y | Y | Y | Y | N | Y | Y | N | Y | 10 |
| Atsuya S.[[19](#_ENREF_19)] | Y | Y | Y | U | Y | Y | N | Y | Y | N | Y | Y | Y | Y | 11 |
| Ishii M.[[20](#_ENREF_20)] | Y | Y | Y | Y | Y | Y | Y | Y | Y | N | Y | Y | U | Y | 12 |

**Supplementary Table2: QUADAS assessment of included articles**

Item 1: Was the spectrum of patients representative of the patients? Item 2: Were selection criteria clearly described? Item 3: Isthe reference standard likely to classify the target condition? Item 4: Is the time period between reference standard and indextest short enough? Item 5: Did the whole sample use a reference standard of diagnosis? Item 6: Did patients receive the samereference standard regardless of the index test result? Item 7: Was the reference standard independent of the index test? Item8: Was the index test described in sufficient detail? Item 9: Was the reference standard described in sufficient detail? Item 10:Were the index test results interpreted without knowledge of the results of thereference standard? Item 11: Were the referencestandard results interpreted without knowledge of the results of the index test? Item 12: Were the same clinical data availablewhen test results were interpreted as would be available when the test is used in practice? Item 13: Were uninterpretable/intermediate test results reported? Item 14: Were withdrawals from the study explained? [[21](#_ENREF_21)]

Y: yes; N: no; U: unclear

**References**

1. Feng X, Song P, Bie P, Jiang P, Ma K, Li X, Wang S, Wang Z, Tang W, Zheng S. Des-gamma-Carboxyprothrombin Plasma Level in Diagnosis of Hepatocellular Carcinoma in a Chinese Population Undergoing Surgery. Medical Science Monitor. 2016; 22: 1663-72. doi: 10.12659/msm.895483.

2. Jang ES, Jeong SH, Kim JW, Choi YS, Leissner P, Brechot C. Diagnostic Performance of Alpha-Fetoprotein, Protein Induced by Vitamin K Absence, Osteopontin, Dickkopf-1 and Its Combinations for Hepatocellular Carcinoma. PLoS One. 2016; 11: e0151069. doi: 10.1371/journal.pone.0151069.

3. Ji J, Wang H, Li Y, Zheng L, Yin Y, Zou Z, Zhou F, Zhou W, Shen F, Gao C. Diagnostic Evaluation of Des-Gamma-Carboxy Prothrombin versus alpha-Fetoprotein for Hepatitis B Virus-Related Hepatocellular Carcinoma in China: A Large-Scale, Multicentre Study. Plos One. 2016; 11. doi: 10.1371/journal.pone.0153227.

4. Lim TS, Kim DY, Han K-H, Kim H-S, Shin SH, Jung KS, Kim BK, Kim SU, Park JY, Ahn SH. Combined use of AFP, PIVKA-II, and AFP-L3 as tumor markers enhances diagnostic accuracy for hepatocellular carcinoma in cirrhotic patients. Scandinavian Journal of Gastroenterology. 2016; 51: 344-53. doi: 10.3109/00365521.2015.1082190.

5. Seo SI, Kim HS, Kim WJ, Shin WG, Kim DJ, Kim KH, Jang MK, Lee JH, Kim JS, Kim HY, Kim DJ, Lee MS, Park CK. Diagnostic value of PIVKA-II and alpha-fetoprotein in hepatitis B virus-associated hepatocellular carcinoma. World Journal of Gastroenterology. 2015; 21: 3928-35. doi: 10.3748/wjg.v21.i13.3928.

6. Song P, Feng X, Inagaki Y, Song T, Zhang K, Wang Z, Zheng S, Ma K, Li Q, Kong D, Wu Q, Zhang T, Zhao X, et al. Clinical utility of simultaneous measurement of alpha-fetoprotein and des-gamma-carboxy prothrombin for diagnosis of patients with hepatocellular carcinoma in China: A multi-center case-controlled study of 1,153 subjects. Biosci Trends. 2014; 8: 266-73. doi:

7. Ertle JM, Heider D, Wichert M, Keller B, Kueper R, Hilgard P, Gerken G, Schlaak JF. A Combination of alpha-Fetoprotein and Des-gamma-Carboxy Prothrombin Is Superior in Detection of Hepatocellular Carcinoma. Digestion. 2013; 87: 121-31. doi: 10.1159/000346080.

8. Truong BX, Yano Y, Van VT, Seo Y, Nam NH, Trach NK, Utsumi T, Azuma T, Hayashi Y. Clinical utility of protein induced by vitamin K absence in patients with chronic hepatitis B virus infection. Biomed Rep. 2013; 1: 122-8. doi: 10.3892/br.2012.4.

9. Hady AA, El Shanawany F, Hassan MM, Anas A, Mostafa I, Hadi AA. Evaluation of Human Telomerase Activity as a Novel Tumor Marker for Hepatocellular Carcinoma. Life Science Journal-Acta Zhengzhou University Overseas Edition. 2010; 7: 153-61. doi:

10. Sharma B, Srinivasan R, Chawla YK, Kapil S, Saini N, Singla B, Chakraborthy A, Kalra N, Duseja A, Dhiman RK. Clinical utility of prothrombin induced by vitamin K absence in the detection of hepatocellular carcinoma in Indian population. Hepatology International. 2010; 4: 569-76. doi: 10.1007/s12072-010-9186-2.

11. Marrero JA, Feng Z, Wang Y, Nguyen MH, Befeler AS, Roberts LR, Reddy KR, Harnois D, Llovet JM, Normolle D, Dalhgren J, Chia D, Lok AS, et al. alpha-Fetoprotein, Des-gamma Carboxyprothrombin, and Lectin-Bound alpha-Fetoprotein in Early Hepatocellular Carcinoma. Gastroenterology. 2009; 137: 110-8. doi: 10.1053/j.gastro.2009.04.005.

12. Yoon YJ, Han K-H, Kim DY. Role of serum prothrombin induced by vitamin K absence or antagonist-II in the early detection of hepatocellular carcinoma in patients with chronic hepatitis B virus infection. Scandinavian Journal of Gastroenterology. 2009; 44: 861-6. doi: 10.1080/00365520902903034.

13. Volk ML, Hernandez JC, Su GL, Lok AS, Marrero JA. Risk factors for hepatocellular carcinoma may impair the performance of biomarkers: a comparison of AFP, DCP, and AFP-L3. Cancer biomarkers : section A of Disease markers. 2007; 3: 79-87. doi:

14. Nakamura S, Nouso K, Sakaguchi K, Ito YM, Ohashi Y, Kobayashi Y, Toshikuni N, Tanaka H, Miyake Y, Matsumoto E, Shiratori Y. Sensitivity and Specificity of Des-Gamma-Carboxy

Prothrombin for Diagnosis of Patients with Hepatocellular

Carcinomas Varies According to Tumor Size. The American journal of gastroenterology. 2006; 101: 2038-43. doi: 10.1111/j.1572-0241.2006.00681.x.

15. Wang C-S, Lin C-L, Lee H-C, Chen K-Y, Chiang M-F, Chen H-S, Lin T-J, Liao L-Y. Usefulness of serum des-gamma-carboxy prothrombin in detection of hepatocellular carcinoma. World journal of gastroenterology. 2005; 11: 6115-9. doi:

16. Marrero JA, Su GL, Wei W, Emick D, Conjeevaram HS, Fontana RJ, Lok AS. Des-gamma carboxyprothrombin can differentiate hepatocellular carcinoma from nonmalignant chronic liver disease in american patients. Hepatology. 2003; 37: 1114-21. doi: 10.1053/jhep.2003.50195.

17. Cui R, Wang B, Ding H, Shen H, Li Y, Chen X. Usefulness of determining a protein induced by vitamin K absence in detection of hepatocellular carcinoma. Chinese medical journal. 2002; 115: 42-5. doi:

18. Ikoma J, Kaito M, Ishihara T, Nakagawa N, Kamei A, Fujita N, Iwasa M, Tamaki S, Watanabe S, Adachi Y. Early diagnosis of hepatocellular carcinoma using a sensitive assay for serum des-gamma-carboxy prothrombin: a prospective study. Hepatogastroenterology. 2002; 49: 235-8. doi:

19. Shimizu A, Shiraki K, Ito T, Sugimoto K, Sakai T, Ohmori S, Murata K, Takase K, Tameda Y, Nakano T. Sequential fluctuation pattern of serum des-gamma-carboxy prothrombin levels detected by high-sensitive electrochemiluminescence system as an early predictive marker for hepatocellular carcinoma in patients with cirrhosis. International journal of molecular medicine. 2002; 9: 245-50. doi:

20. Ishii M, Gama H, Chida N, Ueno Y, Shinzawa H, Takagi T, Toyota T, Takahashi T, Kasukawa R. Simultaneous measurements of serum alpha-fetoprotein and protein induced by vitamin K absence for detecting hepatocellular carcinoma. South Tohoku District Study Group. The American journal of gastroenterology. 2000; 95: 1036-40. doi:

21. Whiting P, Rutjes AW, Reitsma JB, Bossuyt PM, Kleijnen J. The development of QUADAS: a tool for the quality assessment of studies of diagnostic accuracy included in systematic reviews. BMC Med Res Methodol. 2003; 3: 25. doi: 10.1186/1471-2288-3-25.
